# 经支气管镜针吸活检联合现场细胞学对肺癌诊断的临床价值

**DOI:** 10.3779/j.issn.1009-3419.2014.03.06

**Published:** 2014-03-20

**Authors:** 凯述 李, 明涛 刘, 淑娟 姜, 修河 欧阳, 新军 李, 颖 张, 燕燕 李, 柏成 李

**Affiliations:** 1 256610 滨州，山东省滨州市人民医院呼吸内科 Department of Respiratory Medicine, the People's Hospital of Binzhou City, Binzhou 256610, China; 2 250021 济南，山东大学附属省立医院呼吸内科 Department of Respiratory Medicine, Provincial Hospital Affiliated to Shandong University, Jinan 250021, China; 3 256610 滨州，山东省滨州市人民医院病理科 Department of Pathology, the People's Hospital of Binzhou City, Binzhou 256610, China

**Keywords:** 经支气管镜针吸活检, 现场细胞学, 肺肿瘤, Transbronchial needle aspiration (TBNA), Rapid on-site evaluation (ROSE), Lung neoplasms

## Abstract

**背景与目的:**

国内外目前已有多篇现场细胞学应用于经支气管镜针吸活检（transbronchial needle aspiration, TBNA）的报道，但专门针对肺癌患者的研究很少。本研究的目的是探讨TBNA联合现场细胞学在肺癌诊断中的作用。

**方法:**

回顾性分析2012年12月-2013年12月在滨州市人民医院行TBNA并最终确诊为肺癌的69例患者，其中行现场细胞学者37例，未行现场细胞学者32例。比较现场细胞学结果与最终HE染色结果的一致性，同时比较两组患者的诊断率、每个淋巴结穿刺针数、并发症发生率及细胞学诊断费用。

**结果:**

现场细胞学与HE染色一致性为94.1%（32/34）。现场细胞学组诊断率与非现场细胞学组相比无统计学差异（91.9% *vs* 78.1%, *P*=0.20）；但平均穿刺针数及并发症发生率，现场细胞学组少于非现场细胞学组（*t*=29.5, *P* < 0.05; *χ*^2^=4.4, *P* < 0.05），现场细胞学组患者细胞学诊断费用低于非现场细胞学组（*t*=10.9, *P* < 0.05）。

**结论:**

TBNA联合现场细胞学诊断肺癌，与HE染色一致性好，且能减少穿刺针数及并发症，节省细胞学诊断费用，值得推广。

## Abstract (en)

**Background and objective:**

There have been several studys about transbronchial needle aspiration (TBNA) combined with rapid on-site evaluation (ROSE) so far at home and abroad, yet few studys were especially for patients with lung cancer. The aim of our study is to investigate the effect of TBNA combined with ROSE in the diagnosis of lung cancer.

**Methods:**

The data of the patients from December 2012 to December 2013, who were performed with TBNA and ultimately diagnosed with lung cancer in the People's Hospital of Binzhou City, were retrospectively analyzed. The patients were divided into two groups, ROSE group (rapid on-site evaluation group) and no-ROSE group (the group without rapid on-site evaluation). Among these patients, 37 patients were in the ROSE group and 32 patients were in the no-ROSE group. The result of ROSE and HE stain, the diagnostic yields and needle passes of each lymph node, the complication and cytology diagnostic cost of TBNA with ROSE and without ROSE were compared.

**Results:**

The coherence of ROSE and HE stain was 94.1% (32/34). The diagnostic yields of TBNA were 91.9% (34/37) and 78.1% (25/32) in ROSE group and no-ROSE group respectively, no significant differences were found. But the median number of needle passes of each lymph node and the percentage of the complication in ROSE group and no-ROSE group was significantly lower (*t*=29.5, *P* < 0.05 and *χ*^2^=4.4, *P* < 0.05, respectively). The cytopathological diagnostic cost of ROSE group was significantly lower compared with no-Rose group (*t*=10.9, *P* < 0.05).

**Conclusion:**

TBNA combined with ROSE has good concordance with HE stain in the diagnosis of lung cancer, and could reduce the needle passes, cytopathological diagnostic cost and complication, worthy of popularized.

在临床工作中经常遇到肺内占位合并肺门和/或纵隔淋巴结肿大，以及单纯肺门和/或纵隔淋巴结肿大患者，这种情况下单纯依靠胸部CT不能确诊，因为很多疾病可以有类似影像学表现，如肺癌、转移性肿瘤、淋巴瘤、结节病、淋巴结结核等。支气管镜粘膜活检或刷检能明确镜下有新生物或粘膜有浸润表现的部分患者，但对镜下无新生物及粘膜正常的患者，常规支气管镜检查却难以明确诊断。这种情况往往需要依靠经支气管镜针吸活检（transbronchial needle aspiration, TBNA）、超声支气管镜引导下经支气管镜针吸活检（endobronchial ultrasound-guided transbronchial needle aspiration, EBUS-TBNA）或纵隔镜才能明确诊断。其中纵隔镜仍是目前纵隔淋巴结活检的“金标准”^[1]^。但标准纵隔镜在很多地方不能常规开展，且需要在全麻下进行、费用昂贵、需要住院，并发症和死亡率在1.4%-2.3%^[2]^，目前往往在TBNA和EBUS-TBNA结果阴性时才考虑行纵隔镜。由于经济因素，国内开展EBUS-TBNA的医院很少，因此在大多数医院TBNA仍是明确纵隔或肺门肿大淋巴结性质的首选方法。

由于TBNA系“盲穿”，操作时不能直接看到气管或支气管壁外的结构^[3]^，因此操作过程中不能确定是否穿刺到目标淋巴结。很多临床医生由于阳性率低或害怕由此引起的风险而放弃该项检查。为了解决这个问题，现场细胞学（rapid on-site evaluation, ROSE）开始应用于临床^[4]^。现场细胞学检查是在支气管镜检查过程中，由细胞病理学家现场对穿刺标本进行制片和染色，并进行快速评价，向操作者反馈穿刺是否成功，并提供初步诊断的一种方法^[5]^。国内外目前已有多篇^[4, 6-10]^现场细胞学用于TBNA的报道，但专门针对肺癌患者的研究很少。我们对行TBNA并最终确诊为肺癌的患者进行回顾性分析，旨在对TBNA联合现场细胞学在肺癌诊断中的作用进行评价。

## 1 研究对象与方法

### 1.1 对象

2012年12月-2013年12月共有74例患者在滨州市人民医院支气管镜室行TBNA，最终诊断为肺癌的69例患者纳入本研究。全部患者胸部强化CT示至少有1组纵隔或肺门淋巴结肿大（淋巴结短经大于10 mm）伴有或不伴有肺内肿块，但支气管镜检查镜下未见明显新生物及肿瘤浸润改变。

### 1.2 TBNA及现场细胞学检查过程

根据第七版肺癌TNM分期系统^[11]^和WANG氏分区定位法^[12]^明确穿刺淋巴结的位置。麻醉成功后，用标准的可弯曲支气管镜（BF-260，奥林巴斯），按文献所述方法^[13, 14]^以王氏122针（Conmed Corprotion, USA）行TBNA，优先选择最易穿刺到的淋巴结，如第7组和4R组淋巴结。每次穿刺完毕后取出穿刺针，并将抽得穿刺液快速打到载玻片上，快速推片。行现场细胞学者由细胞病理学医师以甲苯胺蓝按文献^[4]^所述方法进行染色并现场阅片，如现场细胞学检查结果阳性（图 1），则停止TBNA操作，如检查结果阴性则由术者在原部位或另选穿刺点再次穿刺，每个部位穿刺次数最多不超过7次^[15]^，操作结束后所有涂片送病理科脱色后行HE染色，由2名高年资病理科医师阅片并确定最终诊断，作为“金标准”。不行现场细胞学者穿刺结束所有涂片置95%乙醇固定后常规送HE染色。非现场细胞学组按文献^[16, 17]^推荐穿刺3针-5针，由操作者根据穿刺抽吸的效果决定。全部69例TBNA操作由同一名医师完成，操作全程监测心电、血压、指脉氧。

**1:**
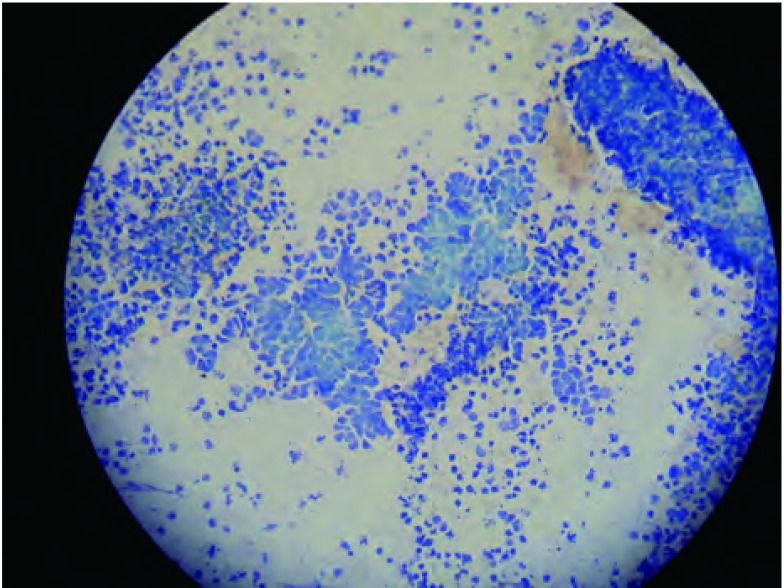
现场细胞学甲苯胺蓝染色（×200）肿瘤细胞胞浆稀少，呈裸核状，核深染，不规则（小细胞肺癌）。 Rapid on-site evaluation with Toluidine blue stain (×200). Tumor cells with little cytoplasm, naked nucleus, and the nucleus were hyperchromatic and irregular (small cell lung caner).

### 1.3 TBNA结果判读

涂片中如果可见多个淋巴细胞团，或较多癌细胞，认为TBNA穿刺成功；如果为大量红细胞或有核细胞很少，则认为TBNA穿刺失败。如涂片中发现癌细胞则认为穿刺结果阳性，如涂片中未发现癌细胞，则认为穿刺结果阴性。每例患者任何一个部位TBNA结果阳性，则认为TBNA总结果阳性；全部部位TBNA结果阴性，则认为TBNA总结果阴性^[18]^。

### 1.4 现场细胞学组与非现场细胞学组相关指标比较

比较现场细胞学结果与最终的HE染色结果的一致性，同时比较TBNA在现场细胞学组及非现场细胞学组的诊断率（现场细胞学组诊断率以HE染色结果为准）、每个淋巴结穿刺针数、并发症发生率及细胞病理学诊断费用。TBNA最常见的并发症为出血，出血量由支气管镜医师根据镜下表现估计。按文献所述^[19]^分为极少量出血、轻度出血、中度出血及重度出血。极少量出血定义为 < 5 mL，轻度出血定义为5 mL-20 mL，中度出血定义为 > 20 mL-100 mL，重度出血定义 > 100 mL。极少量出血不列为并发症范畴。

### 1.5 统计学分析

以SPSS 17.0统计软件进行统计学分析。计量资料采用*t*检验，组间率的比较采*χ*^2^检验，以*P* < 0.05认为有统计学差异。

## 2 结果

### 2.1 患者一般资料及TBNA穿刺淋巴结分布

结果见表 1、表 2。现场细胞学组37例患者中，男25例，女12例，年龄最大76岁，最小35岁，平均（56.8±11.2）岁，肿大淋巴结的短径为（1.8±0.2）cm。非现场细胞学组32例患者中，男性22例，女性10例，年龄最大74岁，最小38岁，平均（57.5±10.8）岁，肿大淋巴结的短径为（1.7±0.5）cm，上述资料两组相比差异无统计学意义。

**1:** 患者一般资料 General data of patients

Variables	ROSE group	No-ROSE group	*P*
Age (yr)	56.8±11.2	57.5±10.8	0.8
Sex (male/female)	25/12	21/11	0.9
Short axis of lymph node (cm)	1.8±0.2	1.7±0.5	0.3
ROSE: rapid on-site evaluation; No-ROSE group: group without rapid on-site evaluation.			

**2:** 淋巴结分布 Location of lymph nodes

Lymph node station	ROSE group (*n*)	No-ROSE group (*n*)
10R	4	1
10L	2	3
11R	2	1
11L	1	0
4L	5	4
4R	8	10
7	22	20
Total	44	39

### 2.2 TBNA穿刺结果

现场细胞学组37例患者共穿刺44个部位，106针，穿刺成功98针（92.5%）。非现场细胞学组32例患者共穿刺39个部位，179针，穿刺成功154针（86.0%）。现场细胞学组37例肺癌患者中，甲苯胺蓝染色共确诊肺癌32例，其中小细胞肺癌16例，鳞癌6例，腺癌10例，其余5例中有2例系现场细胞学阴性，经HE染色证实分别为鳞癌和腺癌，另外3例甲苯胺蓝及HE染色均为阴性，经至上级医院行EBUS-TBNA确诊。非现场细胞学组由TBNA确诊小细胞肺癌12例，鳞癌5例，腺癌8例，其余7例通过经支气管肺活检、EBUS-TBNA确诊，其中鳞癌3例，腺癌4例（表 3）。

**3:** TBNA穿刺结果 Results of TBNA

Diagnosis	ROSE group (*n*=37)^*^		No-ROSE group (*n*=32)^**^
	Toluidine blue stain	HE stain	
Small cell lung cancer	16	16	12
Squamous cell cancer	6	7	5
Adenocarcinoma	10	11	8
Total	32	34	25
^*^Among the 37 patients，there patients got the negative result by TBNA and were diagnosed by EBUS-TBNA; ^**^ Among the 32 patients，two patients were diagnose by transbronchial lung biopsy and five patients were diagnosed by EBUS-TBNA.			

### 2.3 现场细胞学与HE染色一致性

现场细胞学组甲苯胺蓝染色诊断肺癌32例，且均经HE染色证实，未出现假阳性结果，2例现场细胞学检查阴性，经HE染色分别诊断为鳞癌和腺癌，两者一致性为94.1%（32/34）。

### 2.4 两组诊断率对比

现场细胞学组TBNA对肺癌诊断率为91.9%（34/37），非现场细胞学组TBNA诊断率为78.1%（25/32），两者相比差异无统计学意义（*χ*^2^=1.63, *P*=0.20）。

### 2.5 非现场细胞学结果

非现场细胞学组每个淋巴结穿刺针数、镜下出血率、细胞学诊断相关费用均高于现场细胞学组，两者相比有统计学差异（表 4）。其中现场细胞学组和非现场细胞学组分别有4例和10例出现轻度出血，无中度和重度出血及纵隔气肿、纵隔血肿等并发症发生。

**4:** 现场细胞学组与非现场细胞学组相关指标对比 Comparison of index between Rose-group and no-Rose group

	Needle passes (*n*)	Bleeding rate (*n*)	Cytology diagnostic cost (RMB)
No-ROSE group (*n*=32)	4.6±0.4	10/32	140.3±4.6
Rose group (*n*=37)	2.4±0.2	4/37	130.8±2.5
	*t*=29.5	*χ*^2^=4.4	*t*=10.9
*P*	*P* < 0.001	*P*=0.035	*P* < 0.001

## 3 讨论

TBNA是一种获取气道壁、肺实质以及邻近支气管树纵隔内病变部位的细胞学、组织学或微生物学标本的技术。有研究^[20]^将此技术应用于可弯曲支气管镜，目前已在临床广泛开展，在纵隔及肺门肿大淋巴结的诊断及鉴别诊断、肺癌的早期诊断和分期、纵隔及管外型病灶的活检等方面发挥着独特的重要作用。国内荣福教授最早开展该项技术，并逐步在全国范围内推广。目前TBNA已经成为肺癌诊断和分期的有力工具，文献^[21]^报道TBNA阳性率20%-90%，很多医师由于阳性率太低而放弃该项检查。为提高穿刺成功率，我院呼吸科自2012年联合病理科引入现场细胞学技术，并取得了较理想的结果，刚刚开展TBNA的医院可借鉴。

行TBNA时每个淋巴结穿刺几次能获得较高的阳性率，文献报道差异较大。有研究^[16, 22]^推荐3次，有研究^[17]^建议当穿刺仅仅是为了明确诊断，且在一个以上淋巴结穿刺取材或有其他的取材手段时，3针较合适，而当穿刺目的是肺癌的分期时应至少穿刺4针-5针。最近一篇系统性回顾中推荐3针-5针^[23]^。但上述研究均不是专门针对肺癌患者。主要针对肺癌患者的一项前瞻性研究^[15]^中发现穿刺4针-7针可获得最好的诊断率，穿刺7次诊断率达到一个平台，再增加穿刺次数阳性率无明显增加。同时该研究中发现现场细胞学组的平均穿刺次数为（2.5±2.0）次，非现场细胞学组穿刺次数为（3.7±1.6）次，两者相比差异有统计学意义，因此得出现场细胞学能减少穿刺针数的结论。我们的研究中现场细胞学组平均穿刺次数为（2.4±0.2）次、非现场细胞学组平均穿刺次数为（4.6±0.4）次，所得结论与上述研究相一致。同时我们的研究中发现因穿刺针数减少，病理科医师阅片张数减少，因此细胞学诊断费用下降，同样因为穿刺针数减少，应用现场细胞学组出血发生率较未应用现场细胞学组减少，这与Trisolini等^[9]^的研究结论一致。

早期的研究^[4, 7, 8]^发现，与未行现场细胞学检查组相比，现场细胞学组获得不满意标本（定义为没有淋巴细胞或诊断性的/恶性肿瘤细胞）的例数减少，对恶性肿瘤的诊断率高，因此推断现场细胞学能提高诊断率。2011年的发表的两项研究^[9, 10]^，主要评估现场细胞学对胸部CT表现为肺门/纵隔淋巴结肿大患者的诊断价值，这些患者未经筛选，既有肺癌患者，也有淋巴瘤、结节病、结核患者。这两项随机对照研究发现应用现场细胞学组与未应用现场细胞学组相比诊断率无统计学差异，因此考虑早期研究结果可能与选择偏倚有关。我们的研究中现场细胞学组诊断率为91.9%，非现场细胞学组诊断率为78.1%，两组诊断率无统计学差异，与上述两项研究结论相一致。本研究与之前研究对比，优势是研究对象是专门针对肺癌患者，潜在缺陷之一是未使用随机对照，因此上述结论尚需专门针对肺癌的大样本、随机对照研究进一步证实。

文献^[24, 25]^报道第4组和第7组淋巴结穿刺阳性率较高，因此我们在穿刺时大多首选这两组淋巴结。本研究最终对肺癌的诊断阳性率（以HE染色为准）为91.9%（34/37），略高于国内文献^[18, 26, 27]^所报道，考虑除与应用现场细胞学有关外，也与所选择穿刺部位多为第4和第7组淋巴结有关。

我们的研究中现场细胞学对小细胞肺癌的诊断率为100%（16/16），对腺癌和鳞癌的诊断率相对偏低，这与李香菊等^[28]^的报道一致。文献^[23]^报道小细胞肺癌穿刺阳性率较高可能是因为小细胞肺癌生物学侵袭性高、黏附性低，且容易侵犯4R组淋巴结。刚开展TBNA的医院可以从临床疑诊小细胞肺癌的患者开始练习穿刺，以提高自信心。通过我们的观察发现，以甲苯胺蓝行现场细胞学与HE染色相比一致性为94.1%（32/34）。有2例患者现场细胞学阴性，而HE染色证实分别为鳞癌和腺癌，这提示我们现场细胞学所提供诊断为临时诊断，最终诊断仍应以常规HE染色为准。

现场细胞学检查需要细胞病理学家的参与，但国内的现状是病理科医生日常工作非常繁忙，很难抽出专门的人员参与现场细胞学检查，因此国内很少有医院开展这项工作。但最近*Chest*上发表的一项研究^[29]^有望改变这种局面。在这项研究中肺科医师经过3个月的细胞病理学知识培训后行现场细胞学检查，准确率可达80%，与细胞病理学家相比（准确率92%）无统计学差异。因此我们有理由相信将来会有越来越多的肺科医师参与现场细胞学检查，这将大大推进这项技术的普及。

总之，TBNA联合现场细胞学诊断肺癌，与HE染色一致性好，且能减少穿刺针数及并发症，节省细胞学诊断费用，值得推广。
